# Identification of Key Factors Influencing the Choice of the Type of Vaginal Pessary for Women Presenting with Pelvic Organ Prolapse: Semi-Directive Interviews and Development of an Algorithm

**DOI:** 10.3390/jcm12041548

**Published:** 2023-02-15

**Authors:** Marie-Amélie Le Quoy, Odile Cotelle, Renaud de Tayrac, Florence Happillon, Antoine Pelhuche, Valérie Wenner-Vidal, Blandine Liagre, Florence Cour, Camille Armengaud, Gautier Chene, Emilie Cerutti, Fabienne Doucet, Anne-Cécile Pizzoferrato, Xavier Deffieux

**Affiliations:** 1 Service de Gynécologie-Obstétrique, Assistance Publique—Hopitaux de Paris (APHP), GHU Paris Saclay, Hopital Antoine Beclere, 157, rue de La-Porte-de-Trivaux, F-92140 Clamart, France; 2 Service de Gynécologie, CHRU Carémeau, rue du Professeur-Debré, CEDEX 9, F-30029 Nîmes, France; 3 Independent Researcher, 35 Bis Route Corzent, F-74200 Anthy sur Léman, France; 4 Unité D’urodynamique, Assistance Publique—Hopitaux de Paris (APHP), GHU Paris Saclay, Hôpital Paul Brousse, 12 Avenue Paul-Vaillant-Couturier, F-94800 Villejuif, France; 5 Laboratoire D’urodynamique, Hôpital Georges Clémenceau, GHU Paris Est, Assistance Publique—Hopitaux de Paris (APHP), 1 rue Georges-Clemenceau, F-91750 Champcueil, France; 6 Independent Researcher, 120 rue Chanzy, F-59260 Hellemmes, France; 7 Service d’Urologie, Hôpital Foch, 40 rue Worth, F-92150 Suresnes, France; 8 Service de Gynécologie-Obstétrique, Centre Hospitalier Intercommunal de Poissy, 10 rue Champ Gaillard, F-78300 Poissy, France; 9 Department of Gynecology, Hôpital Femme Mère Enfant—HFME, University of Lyon, 59 bd Pinel, F-69500 Bron, France; 10 Service d’Urologie, Andrologie et Transplantation Rénale, CHU Jean Minjoz, F-25000 Besancon, France; 11 Service de Gynécologie-Obstétrique, CHU de Poitiers, 2 rue de la Milétrie, F-86000 Poitiers, France; 12 Medecine School, University of Paris Saclay, F-94270 Le Kremlin Bicêtre, France

**Keywords:** pelvic organ prolapse, pessary, algorithm, vaginal pessary, prolapse, decision-making, interview

## Abstract

(1) Background: Pelvic organ prolapse (POP) can be managed using a vaginal pessary. However, the decision-making process whereby health professionals choose the right pessary is unclear. The objective of this study was to focus on the experience of experts in pessary use and to propose an algorithm. (2) Methods: A prospective study, based on face-to-face semi-directive interviews and group discussions, was conducted on a multidisciplinary panel of professional experts specialized in pessary prescriptions. A consensual algorithm was established, and its accuracy was assessed by expert and non-expert panels. The Consolidated Criteria for Reporting Qualitative Studies (COREQ) were used. (3) Results: 17 semi-directive interviews were conducted. The parameters involved in the decision-making process regarding the choice of vaginal pessaries were: desire for self-management (65%), associated urinary stress incontinence (47%), POP type (41%), and POP stage (29%). The algorithm was developed step by step (4 iterations) using the Delphi technique. Most of the expert panel (76%) rated the relevance of the algorithm as 7 or more out of 10 on a visual analog scale according to their own experience (reference activity). Finally, most (81%) of the non-expert panel (*n* = 230) rated the usefulness of this algorithm as 7 or more out of 10 on a visual analog scale. (4) Conclusions: This study provides an expert panel-based algorithm that may help in the prescription of pessaries for POP.

## 1. Introduction

First-line treatment of pelvic organ prolapse (POP) involves the use of a vaginal pessary combined with pelvic floor exercises, whatever the patient’s age and type of prolapse [1,2,3]. A recent Cochrane meta-analysis showed that pessaries, in addition to pelvic floor muscle training, improved women’s POP symptoms and prolapse-specific quality of life [4]. A prospective study also showed that, like surgery [5,6], pessaries improve symptoms (assessed using the PFDI 20 and PFIQ 7 questionnaires) and self-perception of body image (Body Scale Image), regardless of the stage of prolapse [7]. A recent study even showed that long-term use (over one year) of pessaries results in a slight improvement in the stage of prolapse [8]. However, this study had no control group, and it is known that in some cases, regression of prolapse can occur over time, especially in very elderly women because of post-menopausal vulvovaginal atrophy [9].

This reusable medical device inserted into the vagina can be chosen and prescribed by various health professionals (physicians, physical therapists, midwives, etc.). When inserted, the pessary should be comfortable, correct the vaginal bulge and possibly other signs and symptoms (bladder outlet obstruction, overactive bladder, heaviness, etc.) [10], and be easy to remove. Ideally, pessary self-management is the best solution [11], whereby the woman removes and inserts her own pessary.

Many types of vaginal pessaries are available (cube, ring (with or without support), dish (with or without a button), donut, Gellhorn, etc.), but the decision-making process by which health professionals select the appropriate pessary remains unclear. The choice may be influenced by many characteristics of the patient and of the POP. The choice and prescription of pessaries are not currently standardized, and many patients end up with pessaries that are imperfectly adapted to their anatomy, thus leading to poor compliance and abandonment, with the patient often buying several models before finding the right one. It therefore seems important to establish an algorithm to assist health professionals in the choice of pessary and thereby facilitate its prescription and use.

The main objective of this clinical study was to focus on the experience of experts in pessary use, analyze the factors that influence their choice of the type of vaginal pessary, and propose and test the accuracy of an algorithm that helps in the choice of pessary.

## 2. Materials and Methods

This multidisciplinary prospective study was conducted between December 2021 and September 2022. We conducted semi-directive interviews with health professionals who regularly insert pessaries in their everyday practice (called “experts”). The number of participants was fixed according to the principle of data saturation. The Consolidated Criteria for Reporting Qualitative Studies (COREQ) were used [12].

### 2.1. Criteria for Inclusion of Interviewed Health Professionals (Expert Group)

The health professionals (physicians (obstetrician-gynecologists, gynecologists, urologists, general practitioners), physiotherapists, and midwives) included regularly prescribe pessaries. For new patients, and thus for first prescriptions, a threshold of at least one prescription per week (average for the weeks when they are working) was set.

### 2.2. Semi-Directive Interviews

An interview guide was created around several themes (mean duration of the consultation for pessary prescription, mean number of pessaries tried, type of prescription of topical treatments associated with the prescription of pessaries, method of assessing the pessary model and dimensions, precedence of the model or its size, recommendations concerning monitoring after prescription or insertion of the pessary, advice on how often the pessary should be changed, etc.). The characteristics of the participating health professionals were also recorded: age, sex, number of years of experience, profession, type of training in the use of pessaries, professional status (salaried or private), number of consultations per week, surgical activity, mean age and menopausal status of their patients, number of test pessaries available at the doctor’s surgery, mean number of patients seen per week for a first prescription of a pessary, etc. The expert professionals were also asked if they evaluate the dimensions of the pessary to be prescribed using: multiple tests with decreasing sizes, multiple tests with increasing sizes, estimation of the width of the introitus by vaginal examination, or a guesstimate without tests or measurements.

All interviews were conducted by the same investigator (MAL (MD)). They were held face-to-face at the professional’s workplace where possible, or otherwise by videoconference. The interviews were recorded, once the professional’s agreement was obtained, and then transcribed to a computer file. The data were anonymized. The interviews were analyzed according to the principles of content analysis and classified by theme. Each interview lasted around an hour. All participants described their prescribing habits and decision-making process concerning the type of vaginal pessary. The interviews highlighted the parameters used by each health professional during the decision-making process for women presenting with POP. Relevant data were coded and analyzed, and then modeled for the development of an algorithm. The algorithm was developed step by step using the Delphi consensus method, which is a systematic way of assessing the degree of consensus between groups of experts [13]. The process was repeated until a consensus was achieved, ending with four iterations.

### 2.3. Algorithm Development

A first algorithm was established as a function of the parameters used by most professionals in choosing pessaries, and then this algorithm was evaluated by the 17 professionals who participated in the semi-directive interviews. After a first evaluation, a meeting of the professionals was held to discuss the algorithm, and a new algorithm was proposed. Each new algorithm was evaluated by the professionals in terms of its appropriateness for their own expert practice and its usefulness in helping novice professionals to prescribe pessaries. The algorithm was developed step by step using the Delphi technique. The technique consisted of rounds of individual and anonymous questions (online questionnaires and algorithm proposals) to each expert, followed after every round by a group discussion that allowed participants to reflect and adjust their opinions. The process was repeated until a consensus was achieved, ending with four iterations. The algorithm was given a Delphi score (the degree of the professional’s agreement with the algorithm presented) by each professional using a numerical scale from 1 to 10 (1 = complete disagreement; 10 = complete agreement).

### 2.4. External Assessment of the Algorithm

This algorithm was then assessed by an external population of “non-expert” health professionals to determine how helpful they considered it would be in their practice. These professionals were gynecologist-obstetricians with little or no experience in the prescription of pessaries (residents or qualified physicians). A survey by email asked them to assess the usefulness of the algorithm produced by the experts (professionals’ degree of usefulness of the algorithm presented, using a numerical scale from 1 to 10 (1 = not useful/helpful; 10 = very useful/helpful).

### 2.5. Approval by the Ethics Committee

Our study was approved by the French national *Comité d’éthique de la recherche en Obstétrique et Gynécologie* (institutional review board approval number: CEROG 2021-GYN-2017).

## 3. Results

### 3.1. Semi-Directive Interviews

We conducted twenty-one semi-directive interviews with health professionals who practice pessary insertion. Four of these health professionals were excluded because of an inadequate number of pessaries prescribed per week (<1/week), so data were analyzed from seventeen of these professionals, eight of whom were obstetrician-gynecologists, four were physical therapists, two were urologists, one was a midwife, one was a general practitioner, and one was a gynecologist. The characteristics of these professionals are given in Table 1.

Only two professionals held consultations devoted to the prescription and insertion of pessaries. For the others, such consultations were included in their pelviperineology consultations. Appointments for the choice of pessary (prescription or insertion) lasted on average 36 min (±7.8). On average, the health professionals had three pessary models available. Nine professionals reported trying one to two pessaries per patient. Six professionals used three or more pessaries on average. Two professionals did not try the pessaries at appointments but prescribed the chosen model directly to the patient. Twelve out of thirteen professionals considered that there was a steep learning curve in the choice of the pessary best suited to the patient. Most practitioners (70.6%) agreed with the use of topical estrogens if the patient was menopausal. If the patient was not menopausal, practices varied between the prescription of topical estrogens, hyaluronic acid, or other lubricants.

### 3.2. Parameters in the Choice of Pessary

All professionals agreed that the main parameter of choice was the model (before size). In terms of size (dimensions), nine (53%) practitioners reported assessing it approximately by vaginal examination. In the choice of model, the parameters taken into account were, in order of decreasing importance, patient autonomy (desire for self-management of the pessary by the patient) (64.7% of professionals), associated urinary stress incontinence (47.1%), and the type (41.2%) and stage (29.4%) of POP. Other isolated parameters mentioned by a single professional were a large vaginal introitus and the patient’s surgical history. On average, patients were seen again 2.3 months (±0.5) after the first insertion/prescription. In terms of the frequency of pessary change, six practitioners recommended a fixed duration, with an average of 16.8 months (±3.5). The other health professionals recommended changing the pessary as a function of wear and tear (determined by the patient herself or by the professional at follow-up visits).

### 3.3. Method of Evaluation of the Size/Dimensions of the Pessary to Prescribe

When a cube, ring, or donut pessary was chosen, its size was estimated in 10% of cases by trying pessaries of decreasing size, in 20% of cases by trying pessaries of increasing size, in 44% of cases by choosing the size as a function of the width of the vaginal introitus estimated by vaginal examination, and in the remaining cases the professionals reported choosing the size of the cube pessary without trying it and without measurements. Sixty-six percent of the professionals reported that they used vaginal examination to estimate the measurements for the size of the pessary, as a function of the thickness of their fingers (15 to 18 mm); 24% reported that they estimated measurements by vaginal examination using a tape measure graduated in cm, and 10% reported that they used a guesstimate of the measurements.

### 3.4. Development of an Algorithm for the Choice of Pessary

**Figure 1:**
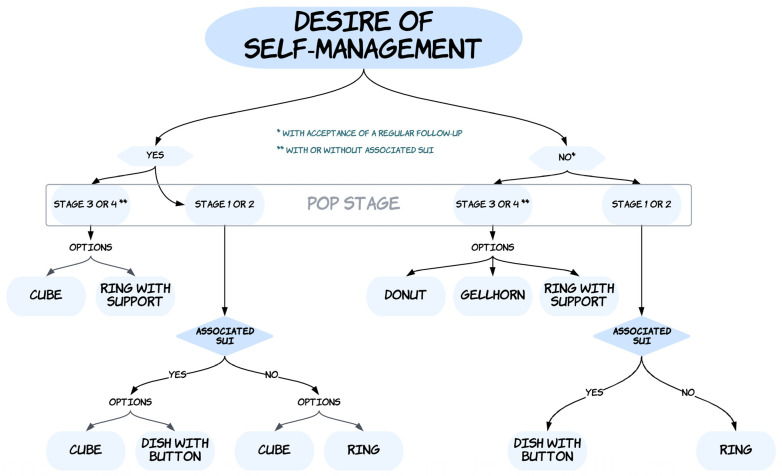
Algorithm for choice of pessary. Abbreviations: POP, pelvic organ prolapse; USI, urinary stress incontinence.

The final algorithm is shown in Figure 1.

Seventy-six percent of the professionals questioned deemed this algorithm to be consistent with their expert practice (degree of agreement of 7 or more on a scale of 1 to 10) and 81% considered (degree of agreement of 7 or more on a scale of 1 to 10) that the algorithm would help novice professionals to choose or prescribe a first pessary for a patient.

The first identified parameter to be considered for pessary choice was the decision of the woman to manage (or not) the daily use of the pessary herself (insertion, removal, cleaning). The second identified parameter for choosing a pessary was the stage of the prolapse (stage 1 or 2 versus stage 3 or 4). When the experts could not decide for a single type of pessary for one situation, they provided “options” (meaning: several pessaries could probably be suitable for the situation, without prioritization between the identified options).

For women wishing to manage their pessaries themselves and presenting with stage 3 or stage 4 POP, two options have been proposed by the experts: either a cube pessary or a ring with support. For women wishing to manage their pessaries themselves and who are presenting with a stage 1 or stage 2 POP, the experts have divided the algorithm according to whether the woman reports an associated USI or not. For those with stage 1 or stage 2 POP associated with USI, the experts offered two options: a cube pessary or a dish pessary with a button. For those with stage 1 or stage 2 POP without an associated USI, the experts offered two options: either a cube pessary or a ring (with or without support).

For women who do not wish to manage their pessaries themselves and present with a stage 3 or stage 4 POP, three options have been proposed by the experts: a donut pessary, a Gellhorn pessary, and a ring with support. For women who do not wish to manage their pessaries themselves and present with a stage 1 or stage 2 POP, the experts have divided the algorithm according to whether the woman reports an associated USI or not. For those presenting with stage 1 or stage 2 POP and an associated USI, experts recommended a dish pessary with a button. For women presenting with stage 1 or stage 2 POP without an associated USI, experts recommended a ring (with or without support).

### 3.5. External Assessment by a Group of Non-Experts of the Usefulness of the Algorithm

Of the members of the non-expert panel (*n* = 230), 81% rated the usefulness of the algorithm as 7 or more out of 10 on a visual analog scale from 1 to 10 where 1 represented “not useful/helpful” and 10 represented “very useful/helpful”.

## 4. Discussion

### 4.1. What Are the Actual Clinical Implications of This Study?

We have determined the parameters used in the choice of pessaries by a panel of experts and have developed a consensual algorithm by questioning experts of various healthcare professions involved in pelviperineology. The semi-directive interviews yielded a wide range of responses, without any leading suggestions on our part. A large panel of non-expert professionals considered our algorithm to be useful in helping with the prescription of pessaries.

Most professionals, including surgeons, think that a pessary can be offered as a first-line treatment in the management of POP [14,15]. However, most health professionals are not comfortable fitting a pessary. An algorithm can therefore help professionals to prescribe a pessary. The use of an algorithm may decrease the number of attempts at fitting pessaries and therefore decrease the cost of management. In most countries (worldwide), there is no reimbursement for vaginal pessaries, and the patient has to pay for each pessary.

### 4.2. What Does This Study Add to the Existing Literature on the Topic?

Such an algorithm is useful because there are few literature reports of algorithms designed to help in the prescription of pessaries. In the current study, the majority of health professionals reported “patient autonomy” as the first parameter to be considered for pessary choice. We defined “patient autonomy” as follows: desire for self-management of the pessary by the patient. The UK guidelines (https://www.ukcs.uk.net/resources/Documents/Pessary%202021/UK%20Pessary%20Guideline%20final%20April21.pdf, accessed on 12 October 2022) also state that, before commencing a pessary fitting, professionals should enquire whether the patient wishes to be sexually active (with vaginal penetration) and to consider self-management (https://www.ukcs.uk.net/resources/Documents/Pessary%202021/Pessary%20choice%20alone.pdf, accessed on 12 October 2022).

In the algorithm presented here, the options among the different clinical POP types are presented as equivalent, without any sequence or preference. For example, “cube or ring with support” pessaries are recommended for women presenting with stage 3 or stage 4 POP and who desire self-management. This is the consequence of the impossibility of achieving a consensus among the experts on these points. Therefore, it is hypothesized that both options are equivalent in these cases. It is seemingly possible to enhance the precision/accuracy of this algorithm in some subgroups. It would be interesting to redo interviews in specific subgroups for which no preference is given in the current algorithm.

The definition of “autonomy” is debatable. However, the preference for self-management is widely prevalent nowadays, as a vaginal pessary is considered a “self-care health product”. We believe that for long-term use, women need to be willing and able to successfully manage (daily removal and cleaning) the pessary. Some women are unable or unwilling to handle vaginal pessaries. Nevertheless, healthcare professionals should propose dedicated consultations to include women in a learning process to help them develop the self-management skills needed to handle pessaries.

The UK guidelines also suggest that the first fitting should be performed with a ring pessary (consider a ring with support for apical prolapse and consider a ring with a knob (button) if urinary stress incontinence is a new (unmasked) or preexisting issue). However, the authors do not state the method used to build this algorithm. Furthermore, one of the main differences between our algorithm and the UK algorithm is that the latter does not consider the stage of POP.

In the instructions for using pessaries, certain algorithms are proposed. For example, in its guide for professionals, the company My Little Pessaire^®^ proposes choosing a model adapted according to three main parameters: the type of POP (cystocele, hysterocele, or rectocele), the presence of urinary stress incontinence (associated with the use of a pessary, unmasked or not), the patient’s wishes concerning the ease of insertion and removal, the patient’s autonomy, and the possibility of sexual activity without removal of the pessary. The other criteria of choice mentioned are a narrow or sensitive introitus, the shape of the pubic arch (shallow/narrow/flat), the absence of a cervix, and the loss of vaginal wall muscle tone. The company Gyneas^®^, which produces and markets pessaries, suggests choosing a pessary as a function of five parameters: POP stage, POP type, associated urinary stress incontinence, a wide vaginal introitus, and sexual activity. The Gyneas^®^ algorithm therefore does not include ease of use, vaginal wall tone, or the presence of a cervix.

To be effective and well tolerated, a pessary should ideally be customized to the patient. Manufacturers offer a panoply of models of different sizes, and 3D printing of customized pessaries seems to be an attractive option for the future [16], though at present 3D modeling of pessaries is at the experimental stage. One company, Femtherapeutics^®^, offers customized 3D printing as a function of parameters entered in its software. These parameters seem to depend solely on clinical data and not on imaging data. However, these parameters are not published or in the public domain.

Lastly, some studies suggest that transperineal ultrasound, by measuring the levator hiatal area, could determine the appropriate pessary size, thereby reducing the risk of pessary expulsion [17]. The objective of this prospective case–control study was to predict the successful ring pessary size based on the levator ani hiatal area measured using 3D/4D transperineal ultrasound. As a result, unsuccessful fitting trials, due to the dislodgment of a small ring pessary, were correlated with the levator ani hiatal area measured by an ultrasound. However, this study only assessed a cut-off level for dislodgment for one type of pessary (a small ring). For now, it is not possible to determine the type and size of the pessary according to ultrasonographic findings. Furthermore, such measurements do not seem to be predictive of pessary failure owing to patient discomfort.

In our interviews, another question elicited highly varied responses: the duration of use of the pessary. Many practitioners recommend changing the pessary at least once a year for preventive reasons [18]. However, a yearly change of pessary economically impacts patients, who must pay for a new pessary at each change. The point is that the frequency of the change probably reflects the type of material which the pessary is made out of (silicon or latex). A silicon pessary may be used for 5 years, since it lasts longer than the latex ones. Further studies are needed to estimate how long a pessary can be used without generating complications.

### 4.3. What Are the Limitations of the Current Study and What Are the Future Study Perspectives?

Our study is subject to some limitations. The size of the expert panel may seem limited, but for highly homogeneous samples (such as niche jobs, i.e., pelviperineology), saturation usually happens after as few as 10 to 20 interviews. Saturation in qualitative research is defined as follows: when, through the course of interviewing, the researcher notices the same themes cropping up repeatedly. In other words, as you interview more and more participants, you stop finding new opinions. This limited number of interviews did not allow us to compare the answers as a function of parameters specific to the interviewees. Despite this, there appear to be similarities in the answers provided by practitioners of the same health profession. Most of the professionals interviewed were obstetrician-gynecologists. Further, it would be interesting to prospectively validate this algorithm in real-life clinical consultations, in expert and non-expert multidisciplinary groups, and in various regions of the world.

To conclude, this study provides an expert panel-based algorithm that may help in the prescription of pessaries for POP.

## Figures and Tables

**Table 1:** Characteristics of the professionals questioned in semi-directive interviews.

**Total**	*n* = 17
**Age in y.** mean (s.d.)	45.8 (12.5)
**Sex:** female *n*.(%)	12 (70.6)
**Years of professional practice** mean (s.d.)	16.9 (8.3)
**Type of practice** *n*.(%)	
Private	5 (29.4)
Salaried	8 (47.1)
Mixed	4 (23.5)
**POP surgical practice: “yes”** *n*.(%)	11 (64.8)
**Training in the prescription of pessaries** *n*.(%)	
Peer training	8 (47.1)
Self-study (books, journals, and internet)	15 (58.8)
Training organized by industry	4 (23.5)
**Mean number of new pessaries prescribed/week** ^1^ *n*.(s.d.)	4.7 (1.4)

^1^ first prescription of a pessary for a new patient. Abbreviations: *n*., number; s.d., standard deviation; y., years.

## Data Availability

Upon request from the corresponding author.
